# Outcomes among patients with *Staphylococcus aureus* bacteremia enrolled in a postdischarge outpatient parenteral antibiotic therapy program at an academic medical center

**DOI:** 10.1017/ash.2022.302

**Published:** 2022-10-10

**Authors:** Deborah A. Theodore, E. Yoko Furuya, Eloise Austin, William G. Greendyke

**Affiliations:** 1 Division of Infectious Diseases, Department of Medicine, Columbia University Irving Medical Center, New York, New York; 2 Infection Prevention and Control, NewYork-Presbyterian Hospital, New York, New York

## Abstract

We compared patients with *Staphylococcus aureus* bacteremia enrolled in outpatient parenteral antibiotic therapy monitoring program (OPAT-MP) upon hospital discharge with patients not enrolled. OPAT-MP patients were more likely to attend infectious diseases follow-up appointments. OPAT-related emergency room visits and/or readmissions were more common among non–OPAT-MP patients, but differences were not statistically significant.

Outpatient parenteral antibiotic therapy (OPAT) has become an integral part of the treatment of deep-seated infections. Advantages of OPAT include cost-effectiveness, decreased exposure to hospital-acquired infections, and improved patient satisfaction; disadvantages include risk of readmission, catheter-related complications, and adverse drug events.^1–5^

A notable indication for OPAT is *Staphylococcus aureus* bacteremia, which requires 2–6 weeks of intravenous antibiotic therapy.^6^ Many patients are therefore discharged from the hospital on intravenous anti-staphylococcal treatment. However, in the absence of appropriate laboratory monitoring, the available antibiotics (eg, vancomycin, cefazolin, and β-lactams such as oxacillin and nafcillin) for *S. aureus* bacteremia are associated with significant and potentially life-threatening side effects.^6^ OPAT monitoring is therefore particularly applicable for this patient group. Given the persistent high morbidity and mortality of *S. aureus* bacteremia,^6^ new strategies to improve clinical outcomes are imperative.

We conducted a retrospective study to investigate whether a structured OPAT monitoring program (OPAT-MP) with dedicated faculty support improved outcomes among patients discharged after an episode of staphylococcal bacteremia.

## Methods

A formal OPAT-MP was established in July 2016 by the Division of Infectious Diseases at Columbia University Irving Medical Center (CUIMC), an academic, multicampus, acute-care hospital center in New York City. As part of the program, dedicated infectious diseases (ID) clinicians review weekly laboratory results collected by home infusion services (1) to adjust antibiotic levels and monitor antibiotic toxicity, (2) to troubleshoot issues regarding long-term intravenous lines to prevent emergency room (ER) visits and hospital readmissions, and (3) to coordinate with the primary ID physician to ensure proper completion of therapy.

We conducted a retrospective chart review of patients discharged home on intravenous antibiotic therapy after a hospital admission at CUIMC for *S. aureus* bacteremia. Patients admitted between July 2016 and December 2017 and enrolled in the OPAT-MP at discharge were compared to those admitted between January 2015 and December 2017 who were not enrolled in OPAT-MP at discharge. Patients discharged to a short- or long-term rehabilitation center or nursing care facility were excluded. Outcomes included ID follow-up appointment attendance, OPAT-related hospital readmissions and ER visits, microbiological recurrences, and death. Readmissions and ER visits were deemed OPAT related if they were due to intravenous catheter complications (including thrombosis, catheter dislodgement, or secondary line-associated bloodstream infections), adverse drug reactions, new *Clostridium difficile* infection, or worsening of the primary infection after source control had previously been achieved.

Statistical measures included χ^2^ tests or Fisher exact tests, as indicated. SAS version 9.4 software (SAS Institute, Cary, NC) was used for all analyses.

This study was approved by the Institutional Review Board of Columbia University Irving Medical Center.

## Results

Overall, 151 patients were discharged on intravenous anti-staphylococcal therapy for *S. aureus* bacteremia during the study period: 45 patients were included in the OPAT-MP cohort and 106 patients were discharged on OPAT without formal monitoring. The median age of patients was 55.8 years and did not significantly differ between the 2 groups (*P* = .34). Sex and race did not significantly differ between groups; more patients in the OPAT-MP group were Hispanic (60% vs 36%; *P* < .01) (Table 1). Chronic kidney disease and end-stage renal disease were more common in the non–OPAT-MP group than in the OPAT-MP group: 43% versus 24% (*P* = .03) and 27% versus 7% (*P* < .01), respectively. Also, 80% of the overall study cohort had an inpatient ID consultation; consultation was more common in the OPAT-MP group than in the unmonitored group (96% vs 74%; *P* < .01). In the OPAT-MP group, at time of bacteremia, Pitt bacteremia scores ranged from 0 to 11 (median score, 1). In the non–OPAT-MP group, Pitt bacteremia scores ranged from 0 to 6 (median score, 1).

**Table 1.** Demographics and Clinical Characteristics of Study Participants

Variable	OPAT-MP Cohort (n = 45), No. (%)^a^	Non–OPAT-MP Cohort (n = 106), No. (%)^a^	*P* Value
Age, median y	53.9	56.3	.3371
Sex, male	24 (53)	67 (63)	.2567
**Race**			.5005
Black/African American	6 (13)	27 (25)	.0784
White	21 (47)	42 (40)	.4420
Other/unknown	18 (40)	37 (35)	.4789
Hispanic	27 (60)	38 (36)	.0081
Diabetes	16 (36)	45 (42)	.4295
Liver disease	4 (9)	11 (10)	1.000
Immunosuppression^b^	11 (24)	25 (24)	.9097
Chronic kidney disease	11 (24)	46 (43)	.0280
End stage renal disease	3 (7)	29 (27)	.0073
Inpatient infectious diseases consultation	43 (96)	78 (74)	.0020
Median duration of admission, d	16.1	16.8	.9024
**MSSA**	32 (71)	79 (75)	.6634
Cefazolin-containing regimen	8 (18)	29 (27)	.2359
Penicillin-containing regimen^c^	23 (51)	39 (37)	.0305
**MRSA**	13 (29)	27 (25)	.6634
Daptomycin-containing regimen	3 (7)	4 (4)	.4258
Vancomycin-containing regimen	10 (22)	23 (22)	1.000
Ceftaroline-containing regimen	1 (2)	2 (2)	1.000
**Source of bacteremia**			
Cellulitis or phlebitis	8 (18)	22 (21)	.6750
Endocarditis	7 (16)	13 (12)	.8562
Osteomyelitis	4 (9)	2 (2)	.0649
Central line	8 (18)	24 (23)	.5036
Pneumonia	3 (7)	4 (4)	.4258
Deep soft tissue infection	9 (20)	20 (19)	.8717
Unknown	2 (4)	12 (11)	.2321
Other	4 (9)	9 (8)	1.000
Lack of source control	4 (9)	13 (12)	.5484
Median planned duration of therapy, weeks	4	3	.4045

Note. OPAT-MP, outpatient parenteral antibiotic therapy monitoring program.

a All values reported as no. (%) unless otherwise specified.

b Immunosuppression: CD4 < 20, prednisone 40 mg daily or greater, solid-organ or bone-marrow transplant, or chemotherapy within 30 d.

c Includes oxacillin, nafcillin, ampicillin-sulbactam, and piperacillin-tazobactam.

**Table 2.** Outcomes

Variable	OPAT-MP Cohort (n = 45)	Non–OPAT-MP Cohort (n = 106)	*P* Value
Attended ID follow-up	25 (56)	40 (38)	.0431
ER visit within 30 d	10 (22)	32 (30)	.1503
OPAT-related	2 (4)	14 (13)	.1096
Readmission within 30 d	12 (27)	33 (31)	.4090
OPAT-related	2 (4)	12 (11)	.1827
Microbiological recurrence	0	2 (2)	.3536
Death ≤30 d	0	0	…
**Therapy outcome**			
Completed as planned	29 (64)	60 (57)	.3704
Stopped early	2 (4)	13 (12)	.2329
Changed or extended	11 (24)	14 (13)	.0893
No documentation	3 (7)	19 (18)	.0729

Note. OPAT-MP, outpatient parenteral antibiotic therapy monitoring program; ID, infectious diseases; ER, emergency room.

The median duration of admission was 16.1 days in the OPAT-MP group and 16.8 days in the unmonitored group. In the OPAT-MP group, 32 patients (71%) had methicillin-sensitive *S. aureus* (MSSA) bacteremia and 13 (29%) had methicillin-resistant *S. aureus* (MRSA) bacteremia. In the non–OPAT-MP group, 79 patients (75%) had MSSA bacteremia and 27 (25%) had MRSA bacteremia. Discharge regimens and sources of bacteremia are described in Table 1. Lack of source control was similar between the OPAT-MP and non–OPAT-MP groups (9% vs 12%, *P* = .55). At the time of discharge, the median planned duration of therapy was 4 weeks for the OPAT-MP group and 3 weeks for the non–OPAT-MP group.

Patients in the OPAT-MP cohort were more likely to attend an ID follow-up appointment (56% vs 38%; *P* = .04) (Table 2). Although OPAT-related ER visits within 30 days of discharge were more common among non–OPAT-MP patients, the difference was not statistically significant (13% vs 4%; *P* = .11). Similarly, although OPAT-related readmissions within 30 days were more common in the non–OPAT-MP cohort, the trend was not statistically significant (11% vs 4%; *P* = .18). Microbiological recurrence within 30 days was low (0% in the OPAT-MP group vs 2% in the non–OPAT-MP group; *P* = .35). There were no deaths in the study cohort within the 30 days after discharge. Most patients completed therapy as planned: 64% in the OPAT-MP cohort and 57% in the non-OPAT-MP cohort (*P* = .37). There was no significant difference between the 2 groups regarding stopping therapy early (*P* = .23), changing or extending therapy (*P* = .08), or lack of documentation of therapy outcome (*P* = .07).

Among the 13 patients in the OPAT-MP cohort with MRSA bacteremia, 4 (31%) required changes in vancomycin dosing over the course of treatment. Of 23 patients in the OPAT-MP cohort with MSSA bacteremia on penicillin-based therapy, 4 (17%) developed drug rash, transaminitis, and/or acute interstitial nephritis. Of these 4 patients, 3 patients required antibiotic therapy changes, and 1 patient was monitored with continuation of therapy.

## Discussion

Among patients discharged on OPAT for *S. aureus* bacteremia, those who were followed by a formal OPAT-MP were significantly more likely to attend an ID follow-up appointment. We detected a nonsignificant trend toward fewer ER visits and readmissions among the patients in the monitored cohort.

This study had several limitations. The sample size was modest. This study was likely underpowered to detect differences in readmissions and ER visits between the 2 cohorts. Many patients who were not enrolled in OPAT-MP had limited follow-up documentation in the medical record after discharge. We detected a bias toward underdetection of adverse drug events and outside hospital readmissions and ER visits in this group. This group also had a higher percentage of patients with chronic or end stage renal disease, which may have predisposed them to having more readmissions or ER visits. Conversely, because patients in the monitored group were followed until the end of their course, they were more likely to have all adverse events captured appropriately.

Although OPAT-related admissions were relatively low, overall readmissions within 30 days approached 30%, indicating that this is a high-risk group. Multiple studies have sought to elucidate the risk factors for readmission among patients on OPAT.^7–9^ In our study, although we did not detect a significant difference in readmissions between the groups, patients in the OPAT-MP group were more likely to attend an ID appointment, and ID follow-up itself has been associated with a lower rate of 30-day readmission among OPAT patients.^10^

Given the advantages of OPAT regarding efficient, effective, and patient-centered care, it is imperative to understand how best to keep patients safe while on OPAT and to further explore the potential benefits of OPAT-MP.
